# Sequential and differential interaction of assembly factors during nitrogenase MoFe protein maturation

**DOI:** 10.1074/jbc.RA118.002994

**Published:** 2018-05-03

**Authors:** Emilio Jimenez-Vicente, Zhi-Yong Yang, W. Keith Ray, Carlos Echavarri-Erasun, Valerie L. Cash, Luis M. Rubio, Lance C. Seefeldt, Dennis R. Dean

**Affiliations:** From the ‡ Department of Biochemistry, Virginia Tech, Blacksburg, Virginia 24061,; the § Department of Chemistry and Biochemistry, Utah State University, Logan, Utah 84322, and; the ¶ Centro de Biotecnología y Genómica de Plantas, Universidad Politécnica de Madrid (UPM), Instituto Nacional de Investigación y Tecnología Agraria y Alimentaria (INIA), Campus Montegancedo UPM Pozuelo de Alarcón, Madrid 28223, Spain

**Keywords:** protein assembly, nitrogenase, reductase, protein purification, electron paramagnetic resonance (EPR), MoFe protein, FeMo-cofactor, P-cluster, nitrogen fixation

## Abstract

Nitrogenases reduce atmospheric nitrogen, yielding the basic inorganic molecule ammonia. The nitrogenase MoFe protein contains two cofactors, a [7Fe-9S-Mo-C-homocitrate] active-site species, designated FeMo-cofactor, and a [8Fe-7S] electron-transfer mediator called P-cluster. Both cofactors are essential for molybdenum-dependent nitrogenase catalysis in the nitrogen-fixing bacterium *Azotobacter vinelandii*. We show here that three proteins, NafH, NifW, and NifZ, copurify with MoFe protein produced by an *A. vinelandii* strain deficient in both FeMo-cofactor formation and P-cluster maturation. In contrast, two different proteins, NifY and NafY, copurified with MoFe protein deficient only in FeMo-cofactor formation. We refer to proteins associated with immature MoFe protein in the following as “assembly factors.” Copurifications of such assembly factors with MoFe protein produced in different genetic backgrounds revealed their sequential and differential interactions with MoFe protein during the maturation process. We found that these interactions occur in the order NafH, NifW, NifZ, and NafY/NifY. Interactions of NafH, NifW, and NifZ with immature forms of MoFe protein preceded completion of P-cluster maturation, whereas interaction of NafY/NifY preceded FeMo-cofactor insertion. Because each assembly factor could independently bind an immature form of MoFe protein, we propose that subpopulations of MoFe protein–assembly factor complexes represent MoFe protein captured at different stages of a sequential maturation process. This suggestion was supported by separate isolation of three such complexes, MoFe protein–NafY, MoFe protein–NifY, and MoFe protein–NifW. We conclude that factors involved in MoFe protein maturation sequentially bind and dissociate in a dynamic process involving several MoFe protein conformational states.

## Introduction

**Figure 1.**
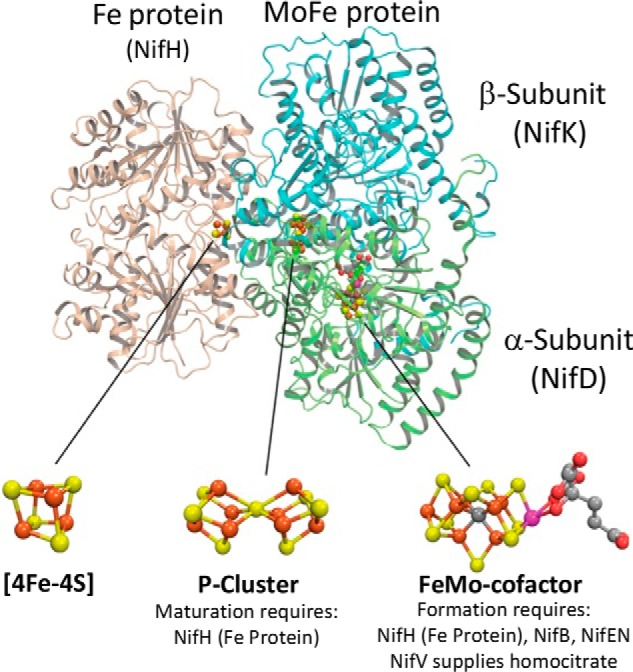
**Schematic representation of molybdenum-dependent nitrogenase and its associated metal-containing cofactors.** Fe protein subunits (encoded by *nifH*) are shown in *light brown*, the MoFe protein α-subunit (encoded by *nifD*) is shown in *green*, and the MoFe protein β-subunit (encoded by *nifK*) is shown in *blue*. Atoms in metal-containing cofactors are indicated as follows: iron (*rust*), sulfur (*yellow*), molybdenum (*magenta*), carbon (*gray*), and oxygen (*red*).

In biology, complex metal-containing enzymes, called nitrogenases, are uniquely capable of catalyzing the nucleotide-dependent reduction of inert atmospheric nitrogen gas (N_2_) to yield fixed nitrogen in the form of metabolically tractable ammonia (NH_3_). There are three genetically distinct, but structurally and mechanistically similar nitrogenases described so far (1–3). These enzymes are differentiated by their primary structures, subunit organizations, and metal composition of their respective active-site cofactors. Among these the best studied is the molybdenum-dependent enzyme, which comprises two catalytic partners, designated the Fe protein and the MoFe protein. Fe protein is a homodimeric nucleotide-dependent switch protein that contains a redox-active [4Fe-4S] cluster located between its identical subunits. The MoFe protein is an α_2_β_2_ heterotetramer harboring two catalytic units and two pairs of complex metal-containing clusters, both of which, so far, are unique in biological systems (4, 5) (Fig. 1). One of these is a [7Fe-9S-Mo-C-homocitrate] species called FeMo-cofactor, and the other is an [8Fe-7S] cluster designated the P-cluster. During each step in the catalytic process, the two component proteins interact to effect nucleotide-dependent, unidirectional, single-electron transfer from the Fe protein to the MoFe protein. Because the nitrogenase-catalyzed reduction of N_2_ requires eight electrons, multiple single-electron-transfer events are required for accumulation of sufficient electrons to effect substrate binding and reduction. For each electron-transfer event the P-cluster mediates inter- and intramolecular electron transfer from the Fe protein to FeMo-cofactor, which provides the substrate activation and reduction site (4, 6). Given the critical importance of the associated metal-containing clusters to nitrogenase catalysis, an understanding of all factors contributing to the robust assembly of MoFe protein containing intact FeMo-cofactor and P-cluster is of considerable interest. Such an understanding is particularly relevant in light of attempts to assemble active nitrogenase components in cereal plants (7, 8).

**Figure 2.**
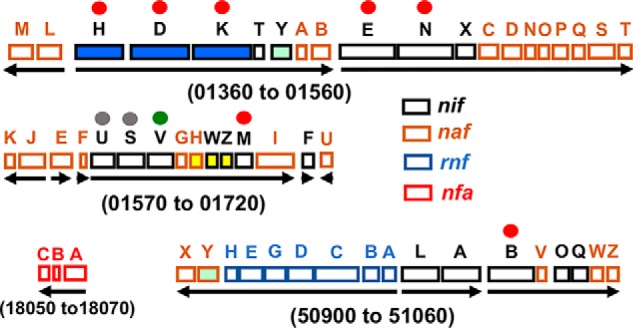
**Organization of the 55 genes associated with the molybdenum-dependent nitrogen fixation system from *A. vinelandii*.** *Numbers* refer to gene designations used in the original annotation of the *A. vinelandii* genome (54), and *arrows* indicate transcription units. The nitrogenase-encoding genes (*nifH* encoding the Fe protein; *nifD* and *nifK* encoding the MoFe protein α and β subunits, respectively) are *filled in blue*. The *nafH*, *nifW*, and *nifZ* genes are *filled in yellow*; and the *nifY* and *nafY* genes are *filled in light green*. The seven genes whose products are strictly required to sustain molybdenum-dependent nitrogen fixation in *A. vinelandii* are indicated by *red dots*. Genes whose products are involved in mobilizing iron and sulfur for nitrogenase-associated metallo-cluster formation are indicated by *gray dots*, and the gene encoding homocitrate synthase, which supplies the organic acid portion of FeMo-cofactor, is indicated by a *green dot*. The gene designations *nif* (nitrogen fixation), *naf* (nitrogenase-associated factor), *rnf* (rhodobacter nitrogen fixation), and *nfa* (nitrogen fixation associated) either have historical origins or have been given formal genetic designations here. Transcriptome analyses have revealed the elevated expression of all of these genes in response to molybdenum-dependent nitrogen-fixing conditions (11, 16). Gene letters do not necessarily indicate similar functions. For example, there is no structural or functional similarity between the products of *nifH* and *nafH.*

The primary translational products of genes encoding the nitrogenase components are not catalytically active. Rather, there is a consortium of associated gene products involved in the assembly and insertion of the necessary metal-containing clusters (9, 10). There are also components that couple cellular metabolism to electron delivery, as well as regulatory elements that control expression of nitrogen fixation associated genes (11–15). In the aerobic, nitrogen-fixing bacterium *Azotobacter vinelandii*, there are 55 genes whose expression is specifically increased in response to a demand for fixed nitrogen when cultured under molybdenum-sufficient conditions (Fig. 2) (11, 16). With respect to formation of fully active MoFe protein, two important features are known. First, FeMo-cofactor is separately assembled and inserted into an inactive form of MoFe protein, often designated apo-MoFe protein (17). Strains inactivated for NifB or NifEN produce FeMo-cofactorless MoFe protein having intact P-clusters (18, 19). NifB is required for formation of a FeMo-cofactor precursor called NifB-co (20, 21). Second, Fe protein, encoded by *nifH* and also referred to as NifH or dinitrogenase reductase, is required not only for nitrogenase catalysis, but also for formation of FeMo-cofactor (22) and for conversion of P-clusters from an immature to a mature form (23). How sulfur and iron are specifically supplied to form P-clusters is not known, but NifS (a cysteine desulfurase) and NifU (an assembly scaffold for formation of simple [2Fe-2S]- and [4Fe-4S]-cluster units) are likely to participate in that process (24). Importantly, mature MoFe protein, MoFe protein produced by a Δ*nifB* strain deficient only in FeMo-cofactor formation, and MoFe protein produced by a Δ*nifH* strain deficient in both FeMo-cofactor formation and P-cluster maturation all exhibit UV-visible and electron paramagnetic spectra that distinguish them from each other (18, 25). These features made it possible to explore how certain aspects of MoFe protein maturation are orchestrated by asking whether particular proteins differentially interact with MoFe protein when captured during various stages of its activation. The construction of strains described here also permitted evaluation of the suggestion that an apo-MoFe protein containing P-cluster precursors, but not FeMo-cofactor, has the capacity for Fe protein-dependent substrate reduction (26).

## Results and discussion

### Spectroscopic features of MoFe proteins purified using a Strep-tagged affinity purification method

**Figure 3.**
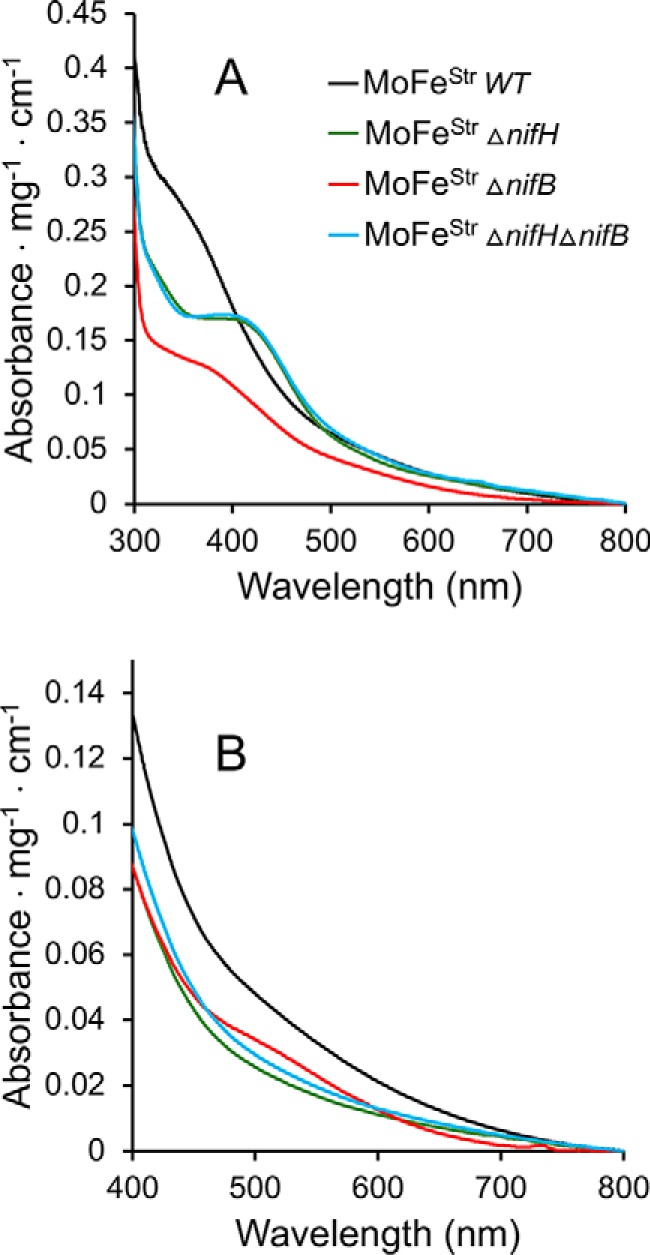
**UV-visible spectra of Strep-tagged MoFe protein prepared from different genetic backgrounds.** Samples in *A* were prepared in the absence of the reducing agent Na_2_S_2_O_4_, and samples in *B* were prepared in the presence of 2 mm Na_2_S_2_O_4_. Spectra in *A* could be reversibly converted to the spectra shown in *B* by the addition of excess Na_2_S_2_O_4_, and spectra in *B* could be reversibly converted to the spectra shown in *A* by the addition of an excess of the oxidizing reagent indigo disulfonate. All proteins were purified using the Strep-tag affinity method.

**Figure 4.**
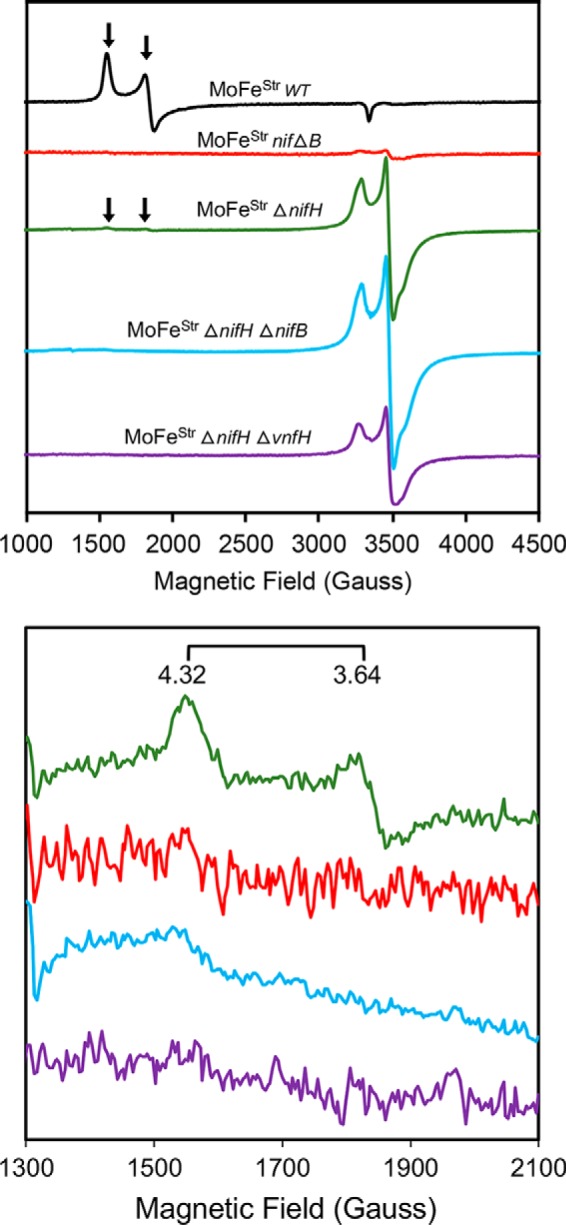
**X-band EPR spectra of resting state Strep-tagged MoFe proteins purified from different *A. vinelandii* strains.** *Black trace*, Strep- tagged MoFe protein produced by WT; *red trace*, Strep-tagged MoFe protein produced by a Δ*nifB* strain; *green trace*, Strep-tagged MoFe protein produced by a Δ*nifH* strain; *blue trace*, Strep-tagged MoFe protein produced by a Δ*nifH* Δ*nifB* strain; *purple trace*, Strep-tagged MoFe protein produced by a Δ*nifH* Δ*vnfH* strain. All samples are Na_2_S_2_O_4_-reduced. The *top panel* shows the full EPR spectrum of each protein with EPR inflections associated with FeMo-cofactor indicated by a *horizontal arrow*. The *bottom panel* shows low-field region spectra, highlighting the EPR signatures (g = 4.32 and 3.64) of the FeMo-cofactor in this region. All spectra were normalized to a final protein concentration of 43.5 μm. EPR parameters are described under “Materials and methods.”

Strep-tag affinity purification methods (27) were used for gentle purification of MoFe protein produced from various genetic backgrounds. In each case, a Strep-tag–encoding sequence was placed at the N terminus of the endogenous *nifD* gene encoding the MoFe protein α-subunit. Strains used are listed in Table 1. Incorporation of a Strep-tag in an otherwise WT strain had no effect on the capacity for diazotrophic growth. Furthermore, affinity purification of Strep-tagged MoFe protein exhibited the same catalytic and spectroscopic features previously reported for MoFe protein having no Strep-tag and purified by other methods (Tables 2 and 3 and Figs. 3 and 4). For these reasons, and for convenience in presentation, Strep-tagged MoFe protein produced in a strain having no other genetic alterations is hereafter referred to as “WT” Strep-tagged MoFe protein unless otherwise indicated. Strep-tagged MoFe protein purified from either a Δ*nifB* or Δ*nifH* strain exhibit UV-visible spectra that differentiate them from each other, and from the WT Strep-tagged MoFe protein (Fig. 3). The spectra of these samples are very similar to those previously reported for corresponding samples prepared by other methods (18, 25). However, the oxidized form of the Strep-tagged MoFe protein prepared from a Δ*nifH* strain exhibits more prominent absorbance in the 400-nm region of the visible spectrum when compared with that reported for the corresponding His-tagged version (25). Whether or not this subtle difference has any significance is not yet clear. The Strep-tagged versions of MoFe proteins produced in this study are otherwise genetically identical to the corresponding and previously characterized His-tagged versions, of which both strain types were constructed in this laboratory (18).

**Table 1:** **Strains used in this study** Strains producing MoFe protein having a Strep-tag placed at the N terminus of the α-subunit are indicated by a superscript Str. Numbers indicate the end points of deletions in genes encoding the relevant gene(s) or the location of amino acid substitution as a result of mutagenesis and gene replacement.

Strain	Genotype
DJ0033	Δ*nifDK* (*nifD*376-*nifK*524)
DJ0035	Δ*nifE* (132–389)
DJ0077	Δ*nifH* (158–201)
DJ0141	Δ*nifB* (60–307)
DJ2102	*nifD*^Str^
DJ2106	*nifD*^Str^ Δ*nifH* (158–201)
DJ2107	*nifD*^Str^ Δ*nifB* (60–307)
DJ2120	*nifD*^Str^ Δ*nifH* (158–201) Δ*nifB*::*Km* (60–307)
DJ2121	*nifD*^Str^ Δ*nifZ* (28–56)
DJ2122	*nifD*^Str^ Δ*nifZ* (28–56) Δ*nifH* (158–201)
DJ2124	*nifD*^Str^ Δ*nifW* (29–86)
DJ2135	*nifD*^Str^ Δ*nifW* (17–63) Δ*nifH* (158–201)
DJ2138	*nifD*^Str^ Δ*nafH* (4–73)
DJ2139	*nifD*^Str^ Δ*nifW* (17–63) Δ*nifZ* (28–56)
DJ2145	*nifD*^Str^ Δ*nifH* (158–201) Δ*vnfH*::*Km* (79–98)
DJ2146	*nifD*^Str^ Δ*nifH* (11–281)
DJ2158	*nifD*^Str^ Δ*nafH* (4–73) Δ*nifH* (158–201)
DJ2165	*nifD*^Str^ 442^Gln^
DJ2195	*nifD*^Str^ 275^Ala^

**Table 2:** **Acetylene reduction features of MoFe proteins prepared from different genetic backgrounds** Fe protein used for these assays was prepared from DJ0033 (Table 1) deleted for the MoFe protein α- and β-subunit–encoding genes to ensure that residual activities could not be attributed to trace levels of contaminating MoFe protein in Fe protein preparations. ND, none detected.

	C_2_H_4_ formed/min^−1^/mg of MoFe protein				
	97% argon, 3% C_2_H_2_	Percentage of WT	97% N_2_, 3% C_2_H_2_	Percentage of WT	Ratio of N_2_/argon
		%		%	
MoFe^Str^ WT	1621.5 ± 74.6	100.0	1001.6 ± 67.0	100.0	0.62
MoFe^Str^ Δ*nifH*	11.8 ± 1.2	0.7	7.3 ± 0.5	0.7	0.62
MoFe^Str^ Δ*nifB*	ND	0.0	ND	0.0	
MoFe^Str^ Δ*nifH* Δ*nifB*	ND	0.0	ND	0.0	
MoFe^Str^ Δ*nifH* Δ*vnfH*	ND	0.0	ND	0.0	

**Table 3:** **Proton reduction features of MoFe proteins prepared from different genetic backgrounds** Fe protein used for these assays was prepared from DJ0033 (Table 1) deleted for the MoFe protein α- and β-subunit–encoding genes to ensure that residual activities could not be attributed to trace levels of contaminating MoFe protein in Fe protein preparations. ND, none detected.

	H_2_ formed/min^−1^/mg of MoFe protein				
	100% argon	Percentage of WT	100% N_2_	Percentage of WT	Ratio of N_2_/argon
		%		%	
MoFe^Str^ WT	2079.0 ± 52.7	100.0	810.7 ± 12.2	100.0	0.39
MoFe^Str^ Δ*nifH*	26.3 ± 5.7	1.3	8.5 ± 4.0	1.0	0.32
MoFe^Str^ Δ*nifB*	ND	0.0	ND	0.0	
MoFe^Str^ Δ*nifH* Δ*nifB*	ND	0.0	ND	0.0	
MoFe^Str^ Δ*nifH* Δ*vnfH*	ND	0.0	ND	0.0	

WT Strep-tagged MoFe protein and Strep-tagged MoFe proteins produced by Δ*nifB* or Δ*nifH* strains also have differentiating spectroscopic signatures when examined by perpendicular mode EPR spectroscopy (Fig. 4). In perpendicular mode EPR, there is an *S* = 32 signature characteristic of FeMo-cofactor (28), an *S* = ½ signature characteristic of immature P-clusters (18, 25), and no characteristic signature for mature P-clusters (18, 28). Consequently, the sequence of MoFe protein maturation could be directly tested using both UV-visible and EPR spectroscopies by comparison of the features of Strep-tagged MoFe protein produced from a strain deleted for both *nifH* and *nifB* with Strep-tagged MoFe protein deleted only for *nifH* or deleted only for *nifB*. Strep-tagged MoFe protein produced from a Δ*nifH* Δ*nifB* strain has an identical UV-visible spectrum (Fig. 3) and a nearly identical EPR spectrum (Fig. 4) when compared with Strep-tagged MoFe protein produced by a Δ*nifH* strain. Small differences in EPR spectra recognized by comparison of Strep-tagged MoFe protein produced by the Δ*nifH* strain and that produced by the Δ*nifH* Δ*nifB* strain is significant and is discussed in detail below. Nevertheless, these comparisons indicate that formation of mature P-clusters precedes insertion of FeMo-cofactor during MoFe protein maturation. This conclusion is in agreement with previous studies that reported Fe protein is necessary to convert MoFe protein produced by a Δ*nifH* strain to the form produced by a Δ*nifB* strain (23, 29).

### Differential and sequential association of assembly factors at different nitrogenase maturation stages

**Figure 5.**
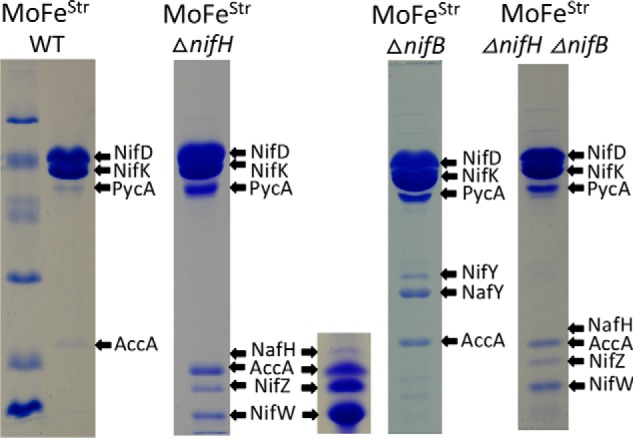
**SDS-PAGE of Strep-tagged MoFe protein samples prepared from various genetic backgrounds using Streptactin affinity columns.** Protein samples shown here and in other figures were separated using a 4% acrylamide stacking gel and 15% acrylamide running gel and then stained with Coomassie Brilliant Blue. Protein standards in the *left panel* include phosphorylase B (97.4 kDa), BSA (66.2 kDa), ovalbumin (45.0 kDa), carbonic anhydrase (31.0 kDa), soybean trypsin inhibitor (21.5 kDa), and lysozyme (14.4 kDa). The *inset* located *adjacent to* the sample showing proteins that co-purify with MoFe protein produced in a Δ*nifH* background represents an overloaded sample to clearly show co-purification of NafH. *Arrows* indicate MoFe protein subunits and co-purifying nitrogen fixation-related proteins and also indicate prominent biotin-binding proteins unrelated to nitrogen fixation, acetate carboxylase subunit (AccA) and pyruvate carboxylase subunit (PycA), that are independently captured by the affinity-purification method. Higher levels of PycA and AccA in certain samples reflect the relatively lower abundance of MoFe protein subunits in extracts of those samples. Very light bands recognized in some samples were identified as various MoFe protein subunit degradation products. All proteins were identified by MS as described under “Materials and methods.” Neither MoFe protein nor any other nitrogen fixation-related protein binds to the Streptactin column if there is no Strep-tag on the MoFe protein.

Denaturing PAGE (SDS-PAGE) revealed that other proteins specifically co-purify with Strep-tagged MoFe protein prepared from Δ*nifH* or Δ*nifB* strains (Fig. 5). In the case of the mature Strep-tagged MoFe protein, there are no co-purifying nitrogen fixation–specific proteins visible by SDS-PAGE. Three proteins, NafH, NifW, and NifZ, co-purify with Strep-tagged MoFe protein samples prepared from a Δ*nifH* strain. Previous studies established that NifW and NifZ are required for formation of a fully active MoFe protein (30, 31), and NifZ has been proposed to be involved in the conversion of immature P-clusters to the mature form (32). No defining physiological or biochemical phenotype has yet been recognized for a Δ*nafH* strain (30). In contrast to Strep-tagged MoFe protein produced by a Δ*nifH* strain, two different proteins co-purify with Strep-tagged MoFe protein produced by a Δ*nifB* strain (Fig. 5). These proteins, NafY and NifY, share primary structure similarity to each other and have been variously proposed to act as “molecular props” that assist FeMo-cofactor insertion or as FeMo-cofactor–trafficking proteins (33–39). These possibilities are not mutually exclusive, and indeed NafY has already been shown to co-purify with apo-MoFe protein produced by a *NifB*-deficient strain (33, 35). NafY also has the capacity to bind FeMo-cofactor (38). Prior work from our laboratory revealed that His-tagged MoFe protein produced in a Δ*nifB* background and purified using an immobilized metal-affinity chromatography (IMAC)^4^ method does not contain any co-purifying proteins (18). This result was interpreted to indicate that high concentrations of imidazole used for sample washing and elution during the IMAC purification procedure might cause dissociation of assembly factors associated with immature MoFe protein, a possibility confirmed in the present work. Purification of Strep-tagged MoFe protein produced by a Δ*nifB* Δ*nifH* strain exhibits the same co-purification of NafH, NifW, and NifZ as recognized for Strep-tagged MoFe protein produced by the singly deleted Δ*nifH* strain (Fig. 5). This observation is in line with the conclusion that P-cluster maturation precedes FeMo-cofactor insertion (40, 41) and indicates that NafH, NifW, and NifZ are dissociated from MoFe protein once P-cluster formation is complete.

### NafY/NifY protect the FeMo-cofactor ligand α-Cys^275^ from rapid alkylation in FeMo-cofactorless MoFe protein produced by a ΔNifB strain

**Figure 6.**
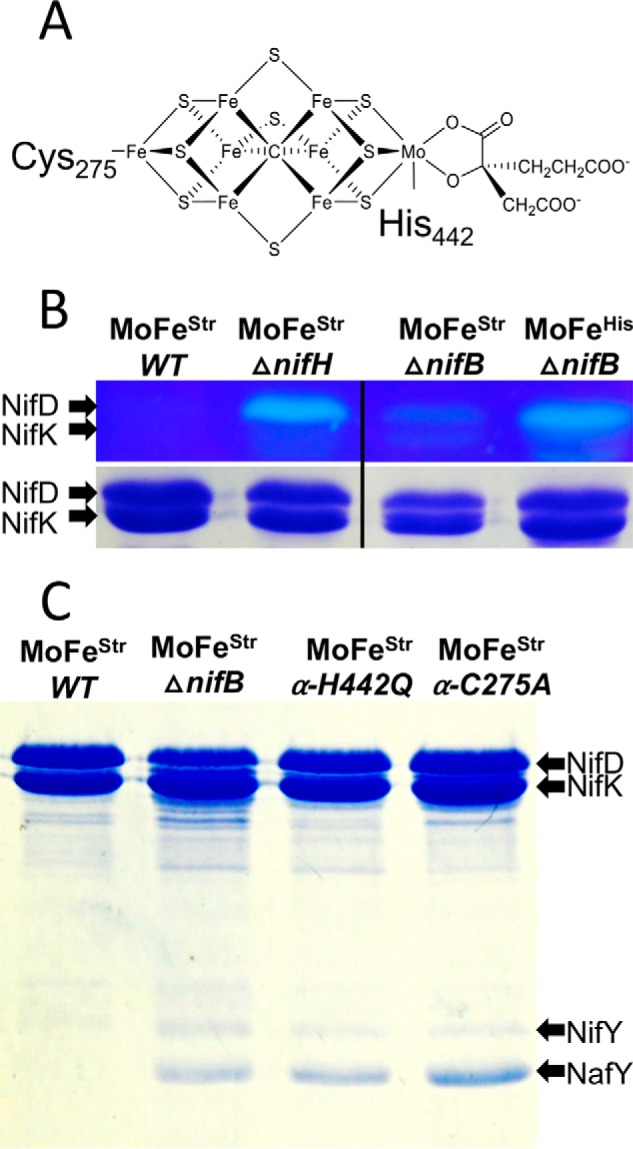
**Susceptibility of the α-Cys^275^ residue of MoFe protein produced in different genetic backgrounds and co-purification of NifY and NafY with MoFe protein having Ala or Gln substituted for either α-Cys^275^ or α-His^442^, respectively.** *A*, structure of FeMo-cofactor showing anchoring α-Cys^275^ and α-His^442^ residues. *B* (*top*), MoFe protein samples purified from different genetic backgrounds and treated with the fluorescent alkylating reagent I-AEDANS before SDS-PAGE and visualized by UV light illumination before Coomassie Brilliant Blue staining (18). Note that *B* is a composite of a single gel for which a lane located between the Δ*nifH* sample and Δ*nifB* sample has been excised. *B* (*bottom*), same samples as shown in the *top* after staining with Coomassie Brilliant Blue. Identities of affinity tags used to assist purification, Strep-tag or His-tag, are indicated by *superscripts. C*, co-purification of NifY and NafY with Strep-tag affinity-purified MoFe protein produced in different genetic backgrounds.

Two residues, α-Cys^275^ and α-His^442^, covalently anchor FeMo-cofactor within the mature MoFe protein (Fig. 6*A*). In the case of apo-MoFe protein purified from Δ*nifB* extracts using the IMAC purification procedure, which has neither NafY nor NifY attached, α-Cys^275^ is hyperreactive to the alkylating reagent [2-(iodoacetamido)ethylamino]naphthalene-1-sulfonic acid (I-AEDANS) (18) (Fig. 6*B*). In contrast, α-Cys^275^ is protected from rapid alkylation in the mature MoFe protein. In the case of NifY/NafY-bound FeMo-cofactorless MoFe purified here using the Strep-tag affinity method, α-Cys^275^ is also protected from rapid alkylation (Fig. 6*B*). This result indicates that either or both NafY and NifY cap the proposed FeMo-cofactor entry site or cause a conformational change, such that the rapid alkylation of α-Cys^275^ by I-AEDANS is denied (Fig. 6*B*). This finding is different from the previously reported alkylation of the α-Cys^275^ residue from FeMo-cofactorless MoFe protein having NafY attached but prepared by a different method (36). In this case, α-Cys^275^ was subject to relatively rapid alkylation, although, in the prior report, a much smaller alkylating reagent, iodoacetic acid, was used compared with the one used in the present work, I-AEDANS, and the time scales used to observe alkylation, minutes *versus* seconds, were also quite different. In contrast to protection of the α-Cys^275^ residue of Strep-tagged MoFe protein produced by a Δ*nifB* strain, α-Cys^275^ is susceptible to rapid alkylation in Strep-tagged MoFe protein produced by a Δ*nifH* strain, which is deficient in both FeMo-cofactor formation and P-cluster activation (Fig. 6*B*). Thus, it appears that the differential susceptibility of α-Cys^275^ to alkylation during different stages of MoFe protein maturation reflects conformational changes and/or differential attachment of maturation factors during the process.

### FeMo-cofactor–coordinating residues α-Cys^275^ and α-His^442^ are required for NafY and NifY dissociation during MoFe protein maturation

No nitrogen fixation–associated proteins are detected by SDS-PAGE that specifically co-purify with Strep-tagged MoFe protein produced by the WT strain. This means that NafY and NifY must dissociate from FeMo-cofactorless MoFe protein upon cofactor incorporation. Such dissociation has been experimentally demonstrated by the simple addition of FeMo-cofactor to a purified FeMo-cofactorless MoFe protein sample having either NifY attached, in the case of experiments using MoFe protein from *Klebsiella pneumoniae* (34), or NafY attached, in the case of MoFe protein from *A. vinelandii* (35). Data shown in Fig. 6*C* reveal that a capacity for covalent attachment by either of the anchoring FeMo-cofactor–anchoring residues, α-Cys^275^ or α-His^442^, is necessary for NafY and NifY dissociation. Namely, both NafY and NifY co-purify with Strep-tagged MoFe protein having either α-Cys^275^ substituted by Ala or α-His^442^ substituted by Gln. Thus, a capacity for covalent attachment to FeMo-cofactor by both anchoring residues is required to lock down a conformation of MoFe protein that promotes dissociation of both NafY and NifY.

### NifY– and NafY–MoFe protein complexes represent two different subpopulations of MoFe proteins specifically deficient in FeMo-cofactor formation

**Figure 7.**
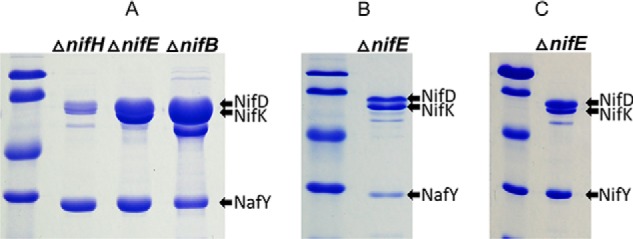
**SDS-PAGE of affinity-purified MoFe protein using immobilized Strep-tagged NafY or Strep-tagged NifY as bait.** Purified Strep-tagged NafY or NifY was immobilized on a Streptactin column, and crude extracts prepared from strains deleted for *nifH*, *nifE*, or *nifB* and expressing MoFe protein that does not have a Strep-tag were separately passed over NafY- or NifY-charged columns, washed with buffer, and eluted using biotin-containing buffer, as described under “Materials and methods.” *A* displays the results of one-step NafY-directed affinity purification of MoFe protein subunits without further processing. The prominent band located *below* NifK in the Δ*nifB* sample is a subunit of the biotin-binding protein pyruvate carboxylase (PycA), which is enriched as a result of the relatively low level of MoFe protein accumulated in crude extracts of that sample. *B* corresponds to the same Δ*nifE* sample shown in *A*, but after further purification to remove excess NafY using gel exclusion chromatography as described under “Materials and methods.” *C* displays the result of one-step NifY-directed affinity purification of MoFe protein subunits without further processing.

MoFe proteins trapped at two fundamentally different stages in the maturation process as a consequence of deletion of either *nifB* or *nifH*, respectively, bind two separate protein sets. However, the relationship among binding proteins associated with a particular assembly stage cannot be deduced from experiments described so far. For example, it was not clear whether or not the associated proteins have the capacity to bind immature MoFe protein forms at the same time or if they represent separate subpopulations. This question was explored with respect to NafY and NifY by using immobilized NafY or immobilized NifY as bait for the affinity purification of apo-MoFe protein produced by strain DJ0035 or DJ0141 deleted for either *nifE* or *nifB*. MoFe proteins produced by DJ0035 and DJ0141 do not carry Strep-tags (Table 1). The results of these experiments revealed that FeMo-cofactorless MoFe protein samples captured by immobilized NafY did not also contain NifY (Fig. 7, *A* and *B*), indicating that NifY and NafY are unlikely to bind at the same time to the FeMo-cofactorless MoFe protein. This conclusion was confirmed by the converse experiment, which showed that FeMo-cofactorless MoFe protein samples captured by immobilized NifY did not also contain NafY (Fig. 7*C*). The order of NafY and NifY binding to immature MoFe protein has not yet been explored.

### Sequential and differential binding of NafH, NifW, and NifZ during MoFe protein maturation

**Figure 8.**
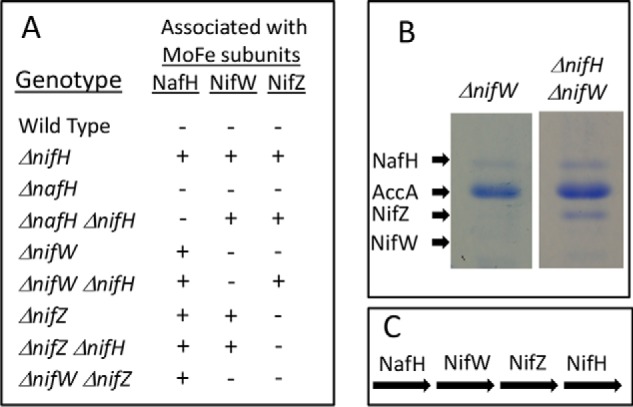
**Sequential and differential interaction of NafH, NifW, and NifZ with MoFe protein during the process of its maturation.** *A*, summary of proteins that co-purify with Strep-tagged MoFe proteins produced in different genetic backgrounds. *B*, an example of experimental results used to generate the data summarized in *A. B*, *first lane*, only NafH co-purifies with Strep-tagged MoFe protein produced by a Δ*nifW* strain (AccA is a subunit of the biotin-binding protein acetate carboxylase, unrelated to nitrogen fixation, that also separately binds to the Streptactin column used for purification). *Second lane*, NafH and NifZ co-purify with Strep-tagged MoFe protein produced by a Δ*nifH* Δ*nifW* strain. Although no NifW is present in these samples, the position of where NifW would migrate on the gel, if present, is indicated. *C*, pathway of NafH, NifW, NifZ, and NifH involvement in MoFe protein maturation deduced from data summarized in *A*.

Similar to the situation of NafY and NifY binding to Strep-tagged MoFe protein produced by a Δ*nifB* strain, the relative binding of NafH, NifW, and NifZ to Strep-tagged MoFe produced by a Δ*nifH* strain is not stoichiometric (Fig. 5). This observation indicated that NafH, NifW, and NifZ might not be members of an individual maturation complex. Instead, the aggregated co-purification of these proteins with Strep-tagged MoFe protein from Δ*nifH* extracts could reflect a mixture of subpopulations of complexes captured at different processing steps leading to MoFe protein having intact P-clusters. Therefore, patterns of the co-purification of NafH, NifW, and NifZ with Strep-tagged MoFe protein produced from different genetic backgrounds deleted, separately and in different combinations, for *nifH*, *nafH*, *nifW*, or *nifZ* were examined (Fig. 8*A*). One example of the results of this experimental approach is shown in Fig. 8*B*. In this case, a Δ*nifW* strain produces Strep-tagged MoFe protein having NafH bound but no NifZ bound. In contrast, Strep-tagged MoFe protein prepared from a Δ*nifW* Δ*nifH* strain has both NafH and NifZ bound. Interpretation of these results is complicated by the fact that deletion of *nifW* slows, but does not eliminate, the capacity for diazotrophic growth (30), suggesting that the function of NifW can be partially supplied by some other protein or that NifW accelerates a process that can occur slowly in its absence. What this means is that Strep-tagged MoFe protein prepared from a Δ*nifW* strain could result in co-purification of any assembly factor that precedes NifW function but not necessarily any that follow its function. In other words, once the function involving NifW is satisfied, MoFe protein is rapidly matured through the action of other intact factors and, therefore, will have no bound assembly factors. Based on this logic, co-purification of NafH with Strep-tagged MoFe protein prepared from a Δ*nifW* strain indicates that NafH interaction must precede NifW interaction (Fig. 8*B*). Furthermore, because NifZ co-purifies with Strep-tagged MoFe protein prepared from a Δ*nifH* strain and from a Δ*nifH* Δ*nifW* strain, the interaction of NifZ occurs after NifW interaction and precedes the NifH-dependent P-cluster maturation step (Fig. 8*C*). The aggregated data using this approach (Fig. 8*A*) lead to a model for the sequential and differential involvement of proteins in the early stages of MoFe protein maturation (NafH-NifW-NifZ-NifH; Fig. 8*C*). The proposed sequential and differential involvement of NafH, NifW, and NifZ in the early stages of MoFe protein maturation mirrors the co-location and co-transcription of their corresponding genes, also in the order *nafH-nifW-nifZ* (Fig. 2).

### Immature MoFe proteins having NafH, NifW, or NifZ bound represent different subpopulations captured in the assembly process

**Figure 9.**
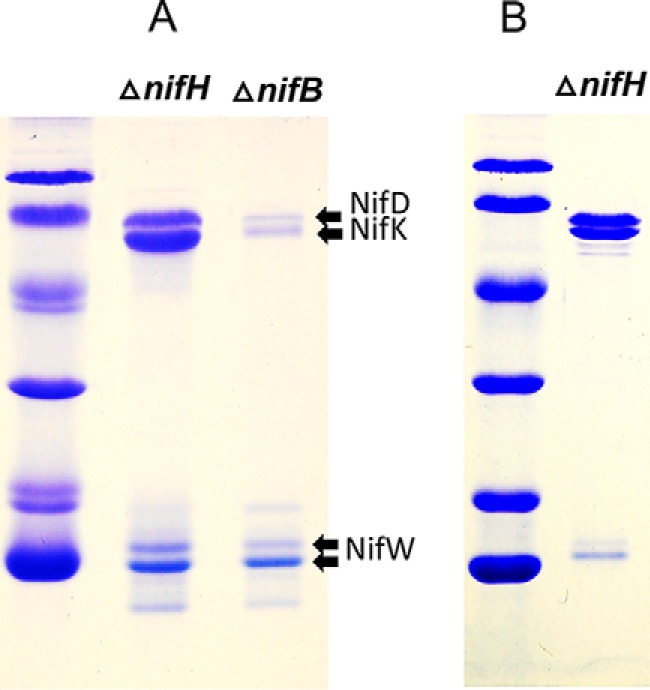
**SDS-PAGE of affinity-purified MoFe proteins using NifW as bait.** Samples were prepared and processed as described under “Materials and methods” as well as in the legend to Fig. 6. *A*, one-step NifW-directed affinity purification of nontagged MoFe protein prepared from extracts of either a Δ*nifH* strain or a Δ*nifB* strain. *B* corresponds to the same Δ*nifH* sample shown in *A*, but after further purification by gel exclusion chromatography. Note that the C terminus of NifW produced in *E. coli* is subject to C-terminal proteolytic cleavage, giving rise to multiple truncated species. This phenomenon has also been previously observed for NifW samples prepared from *A. vinelandii* (42).

Data summarized in Fig. 8 establish that NafH, NifW, and NifZ all have the capacity to bind MoFe protein in the absence of each other and also indicate a temporal order of binding. However, they do not provide insight with respect to their capacity for simultaneous binding. This aspect was addressed in the case of NifW by affinity purification of a specific subpopulation of MoFe protein produced in extracts of a Δ*nifH* strain (DJ0077; Table 1) by using immobilized NifW as bait. Note that MoFe protein produced by DJ0077 does not carry a Strep-tag. Data shown in Fig. 9 reveal capture of a subpopulation of immature MoFe protein from a Δ*nifH* background that has bound NifW but no other co-purifying proteins that can be detected by SDS-PAGE. Passage of extracts prepared from a Δ*nifB* strain (DJ0141; Table 1) using the same NifW-charged column only captures a very small amount of MoFe protein, indicating specificity of the interaction between NifW and MoFe protein produced in a Δ*nifH* background. Capture of a small amount of MoFe protein subunits by NifW when using extracts prepared from NifB-deficient cells is also observed when using WT extracts and could, therefore, indicate that there is a small fraction of MoFe protein having immature P-clusters in such extracts, which could reflect the continuous MoFe protein maturation process in growing cells. This possibility is supported by small inflections in the EPR spectrum associated with immature P-cluster observed in a MoFe protein sample prepared from a Δ*nifB* strain (Fig. 4, *top*, *red trace*). This feature was also recognized in His-tagged MoFe protein prepared from a Δ*nifB* strain using IMAC, which was ascribed to arising either from P-cluster precursors or damaged P-clusters (18). An alternative explanation is that NifW has a low affinity for mature MoFe protein as well as for MoFe protein having intact P-clusters but not FeMo-cofactor. This latter possibility is in line with a previous report indicating interaction between NifW and MoFe protein (42).

### A low level of intact MoFe protein accumulates in a NifH-deficient strain

The capacity for Strep-tagged MoFe protein isolated from either a Δ*nifB* or Δ*nifH* strain to catalyze acetylene and proton reduction is different. Namely, Strep-tagged MoFe protein prepared from a Δ*nifB* background has no detectable acetylene or proton reduction activity, whereas Strep-tagged MoFe protein prepared from a Δ*nifH* background exhibits ∼1–2% of both acetylene and proton reduction capacity compared with WT Strep-tagged MoFe protein (Tables 2 and 3). His-tagged MoFe protein prepared from a Δ*nifH* strain using the IMAC procedure has also been reported to exhibit a low level of acetylene reduction activity (25, 26, 42). One interpretation of these observations is that residual substrate reduction activity exhibited by MoFe protein produced by a Δ*nifH* strain is the result of substrate binding and reduction occurring at a P-cluster “precursor” (26). It has been proposed that such a precursor represents paired [4Fe-4S] clusters separately contained in the respective MoFe protein α- and β-subunits (43). The possibility that Fe protein–dependent acetylene reduction can occur at a P-cluster precursor depends on the assumption that MoFe protein produced by a Δ*nifH* strain contains neither FeMo-cofactor nor intact P-clusters. In support of this possibility, it was reported that MoFe protein prepared from a strain deleted for *nifH* catalyzes the reduction of acetylene as well as reduction of a variety of other artificial nitrogenase substrates, but not reduction of N_2_ (26), ostensibly distinguishing substrate reduction events occurring at either FeMo-cofactor or P-cluster precursors.

Whether or not any Fe protein–directed, ATP-dependent substrate reduction can occur at P-cluster precursors is an important issue because, if so, it gives credence to the suggestion that intact P-clusters might have the capacity for two-electron transfer during catalysis (44). Consequently, a demonstrated *in vivo* ability of Fe protein to direct two-electron chemistry (*e.g.* the nucleotide-dependent two-electron reduction of acetylene to ethylene at P-cluster precursor sites) would have profound implications on models for electron transfer within the nitrogenase complex during catalysis. This issue was therefore examined more carefully.

Detection of NH_3_ at very low levels is unreliable; therefore, conclusions based on the assay used are questionable (45). Because N_2_ is an inhibitor of acetylene and proton reduction catalyzed by the normal nitrogenase (46), such inhibition can be used to gain an indirect assessment of N_2_ binding and, possibly, reduction. As in the case of the WT Strep-tagged MoFe protein, N_2_ is indeed an inhibitor of both acetylene and proton reduction catalyzed by Strep-tagged MoFe produced by a Δ*nifH* strain (Tables 2 and 3). This result suggests, but does not prove, that the residual activity observed for Strep-tagged MoFe protein prepared from the Δ*nifH* strain results from accumulation of a small population of MoFe protein containing intact P-clusters and FeMo-cofactor. These results prompted a more direct and definitive assessment.

If the low level of acetylene and proton reduction observed for Strep-tagged MoFe protein produced by a Δ*nifH* strain does indeed occur at P-cluster precursors, then Strep-tagged MoFe protein produced by a Δ*nifH* Δ*nifB* strain should have the same substrate reduction capacity as Strep-tagged MoFe protein produced by a Δ*nifH* strain. The basis for this assertion is that NifB is exclusively required for FeMo-cofactor formation and has no involvement in P-cluster formation (18). Furthermore, FeMo-cofactor insertion occurs only after, and depends upon, formation of intact P-clusters (40, 41). Strep-tagged MoFe protein prepared from a Δ*nifH* Δ*nifB* strain, however, has no ability to reduce either acetylene or protons (Tables 2 and 3), indicating that the substrate reduction capacity of Strep-tagged MoFe protein produced by the Δ*nifH* strain must require FeMo-cofactor. This conclusion was confirmed by carefully comparing the EPR spectrum of WT Strep-tagged MoFe protein with spectra of Strep-tagged MoFe protein produced by Δ*nifB*, Δ*nifH*, or Δ*nifB* Δ*nifH* strains (Fig. 4). Importantly, Strep-tagged MoFe protein produced by the Δ*nifH* strain exhibits a very minor, but clearly detectable, *S* = 32 EPR signal that is unique to FeMo-cofactor, and this signal is absent in Strep-tagged MoFe proteins prepared from either Δ*nifB* or Δ*nifB* Δ*nifH* backgrounds.

### VnfH can substitute for NifH function in MoFe protein maturation

There are two primary possibilities that could explain how a very small fraction of Strep-tagged MoFe protein produced by a Δ*nifH* strain could contain FeMo-cofactor and intact P-clusters. The *nifH* deletion within the strain used here, and for previous studies, is relatively small, having codons 158–201 deleted (Table 1). It was therefore of interest to test whether this truncated form of Fe protein might retain a slight function in P-cluster maturation and FeMo-cofactor formation. In this respect, it has already been shown that an Fe protein fragment accumulates in extracts of the strain deleted for the *nifH*-encoding residues 158–201 (Fig. 2 in Jacobson *et al.* (30)). A new strain was therefore constructed for which nearly the entire *nifH* region, codons 11–281, are deleted (Table 1). Strep-tagged MoFe protein purified from this strain also retains very low levels of proton and acetylene reduction activities; therefore, an Fe protein fragment cannot be responsible for slight FeMo-cofactor formation and P-cluster maturation (Table 2). The second possibility is that some other protein might supplant the role of Fe protein in P-cluster maturation and FeMo-cofactor formation, albeit at very low levels. Obvious candidates to partially replace NifH function are VnfH or AnfH (47–49), which respectively supply the same catalytic function as NifH for the two other nitrogenase systems. Indeed, it was found that Strep-tagged MoFe protein produced by a strain deleted for both *nifH* and *vnfH* has no capacity for reduction of protons or acetylene (Tables 2 and 3) and exhibits an EPR spectrum having the characteristic *S* = ½ P-cluster precursor signature but no detectable *S* = 32 signature (Fig. 3). This result demonstrates that VnfH is the source of the very low level of P-cluster maturation and FeMo-cofactor formation that can occur in the absence of Fe protein. Thus, a precursor form of the nitrogenase P-cluster does not support Fe protein–dependent reduction of either protons or acetylene at a level that can be detected by GC.

### Conclusions

Work reported here establishes or confirms the following features related to MoFe protein assembly: (*a*) P-cluster maturation precedes FeMo-cofactor insertion; (*b*) two dominant proteins sharing primary structure similarity to each other, NafY and NifY, co-purify with MoFe protein species that have intact P-clusters but do not contain FeMo-cofactor; (*c*) NafY and/or NifY protect the FeMo-cofactor ligating residue α-Cys^275^ from rapid alkylation in MoFe protein lacking FeMo-cofactor but containing intact P-clusters; (*d*) α-Cys^275^ is not protected from rapid alkylation in MoFe protein deficient in both P-cluster maturation and FeMo-cofactor formation; (*e*) dissociation of NafY and NifY from MoFe protein during FeMo-cofactor insertion depends on a capacity for cofactor ligation by both α-Cys^275^ and α-His^442^; (*f*) NafY and NifY are not bound to FeMo-cofactorless MoFe protein at the same time; (*g*) three dominant proteins, NafH, NifW, and NifZ, bind to MoFe protein species deficient in P-cluster maturation and FeMo-cofactor formation; (*h*) NafH, NifW, and NifZ are able to bind immature MoFe protein species in the absence of each other; (*i*) NafH, NifW, and NifZ are sequentially involved in MoFe protein maturation in the order NafH-NifW-NifZ, reflecting the order of transcription of the corresponding *nafH-nifW-nifZ* gene cluster; (*j*) NafH, NifW, and NifZ co-purified with MoFe protein are likely to be associated with separate subpopulations of MoFe protein captured at different substages in P-cluster formation/maturation; (*k*) a subpopulation of MoFe deficient in P-cluster maturation and having only NifW bound can be isolated separately from other maturation complexes by affinity chromatography using NifW as bait; (*l*) the very low level of Fe protein–dependent acetylene and proton reduction activities observed for MoFe protein isolated from a Δ*nifH* strain is not the consequence of substrate reduction occurring at P-cluster precursors; (*m*) the Fe protein counterpart from the V-dependent nitrogenase can substitute at some level for the function of the Fe protein from the molybdenum-dependent system in the maturation of MoFe protein.

The most salient feature of the present work is that sequential binding and dissociation of factors involved in MoFe protein maturation differentiate individual steps in what appears to be a dynamic process involving a variety of conformational states. Demonstration that at least three such immature complexes, MoFe protein–NafY, MoFe protein–NifY, and MoFe protein–NifW, can be separately isolated from all others shows promise for detailed biochemical and structural analysis of individual steps in the maturation of MoFe protein.

## Materials and methods

### A. vinelandii strains

Strains used in this study are listed in Table 1. Defined deletions or missense mutations were incorporated into the *A. vinelandii* genome by congression or marker rescue using transformation of competent cells as described previously (50). Plasmid DNA used for transformation contained known deletions or missense mutations that were created either by restriction enzyme digestion and ligation of parental plasmid DNA containing cloned *A. vinelandii* genomic fragments (9, 30) or by using plasmid vector DNA derived from pUC IDT-AMP containing various synthetic DNA cartridges purchased from Integrated DNA Technologies (Coralville, IA). Strains producing N-terminal Strep-tagged MoFe protein were constructed in the same way as described previously for placement of poly-His–tagged MoFe protein (18), with the exception that the poly-His–encoding sequence was replaced with a Strep-tag–encoding sequence, leading to the N terminal sequence of the endogenously expressed MoFe protein α-subunit: MTGASWSHPQFEK.

### Growth conditions

*A. vinelandii* cells were grown at 30 °C in a 150-liter custom-built fermenter (W. B. Moore, Inc., Easton, PA) in modified Burk medium (51) containing 10 μm Na_2_MoO_4_ as the molybdenum source and 10 mm urea as a nitrogen source. Parameters for growth, derepression of nitrogenase formation, and cell harvesting were the same as described previously (18). *Escherichia coli* strain BL21 (DE3) was used as the host for plasmids pDB2108, pDB2118, and pDB215, which direct the expression of Strep-tagged NifW, Strep-tagged NafY, and Strep tagged NifY, respectively. Plasmids pDB2108, pDB2118, and pdB2115 were constructed by placing the appropriate gene cartridge having the Strep-tag–encoding sequence MASWSHPQFEKH located at the N terminus into the pT7-7 expression vector. Cells harboring pDB2108, pDB2118, or pDB2115 were cultured in 2-liter flasks containing 500 ml of Luria broth medium supplemented with 50 mg of ampicillin in an orbital shaker at 36 °C/300 rpm until they reached *A*_600_ ≈ 0.6. Hyperexpression of the targeted gene was induced by the addition of 5 g of lactose to each 500 ml of culture. After induction, cells were cultured for 3 h at room temperature, harvested by centrifugation, and frozen at −20 °C until used.

### Purification of Strep-tagged MoFe protein

All Strep-tagged versions of MoFe protein produced in various genetic backgrounds were purified using the same protocol. Procedures were carried out anoxically under 100% argon or inside a glove box (Coy Laboratory Products) under 95% N_2_, 5% H_2_ atmosphere. For each purification, 75 g of cells were resuspended in 75 ml of anoxic buffer A (50 mm Tris-HCl, pH 7.9, 20% glycerol, 500 mm NaCl, 2 mm Na_2_S_2_O_4_) containing 0.2 mm phenylmethanesulfonyl fluoride, 2 μm pepstatin, and 1.1 units/ml DNase (Sigma-Aldrich, Darmstadt, Germany). Cell-free extracts were prepared by disruption using a nano-DeBee homogenizer (B.E.E. International, Inc., South Easton, MA) at 25,000 p.s.i. Cell lysates were centrifuged at 58,000 × *g* for 45 min at 4 °C and filtered through a 0.2-μm pore size filter Acrodisc® syringe filter (PALL, Port Washington, NY). Extracts were applied to a 5-ml prepacked Strep-Tactin®XT Superflow® gravity flow column (IBA Lifescience, Göttingen, Germany) equilibrated in anoxic buffer A. The loaded column was washed with 25 ml of buffer A, and samples containing MoFe protein were eluted using buffer A having 50 mm biotin. MoFe protein fractions were concentrated up to <1.5 ml using 100-kDa cutoff pore Amicon® (Darmstadt, Germany) Ultra 0.5-ml centrifugal filters and then buffer-exchanged using a HiTrap^TM^ (GE Healthcare) 5-ml desalting column equilibrated in anaerobic buffer A. Samples used to determine nitrogenase activities were subjected to an additional purification step. In these cases, the buffer-exchanged sample was passed over a 5-ml avidin-agarose (GE Healthcare) gravity column to retain the co-eluted biotin-binding proteins, such as acetate carboxylase, from the previous affinity chromatography step. Protein samples were pelleted and stored in liquid N_2_. Fe protein used for nitrogenase assays was prepared from strain DJ0033 (Table 1) deleted for the *nifD*- and *nifK*-encoding subunits. This ensured that no substrate reduction activities could be attributed to contaminating MoFe protein present in Fe protein samples used for assays. Fe protein was purified as described previously (52).

### Purification of Strep-tagged NafY and Strep-tagged NifW from recombinant E. coli

Cell pellets were resuspended in 3 ml of 50 mm Tris-HCl (pH 7.9), 20 mm EDTA, 0.2 mm phenylmethanesulfonyl fluoride, 2 μm pepstatin, 2 μg/ml DNase for each gram of cells (wet weight), and cell-free extracts were prepared as described above. The cell-free extract was applied to a 5-ml prepacked Strep-Tactin®XT Superflow® gravity flow column (IBA Lifescience) equilibrated with 50 mm Tris-HCl (pH 7.9), 20 mm EDTA (buffer B). The loaded column was washed with buffer B until the eluting fractions exhibited no absorbance at 280 nm. Column-bound proteins were eluted using buffer A having 50 mm biotin and subsequently desalted using a HiPrep 26/10 column (GE Healthcare) equilibrated with buffer B. Samples were frozen at −80 °C until used.

### Affinity purification using Strep-tagged NifW, Strep-tagged NafY, or Strep-tagged NifY as bait

Strep-tagged proteins were immobilized on Strep-Tactin®XT High affinity® (IBA Lifescience) resin by incubation of 40 mg of the appropriate protein with 1 ml of resin for 30 min and then used to prepare a gravity flow column. Excess bait protein was removed by washing with buffer B, and the affinity column was then equilibrated using anoxic buffer C (50 mm Tris-HCl, pH 7.9, 2 mm Na_2_S_2_O_4_, 350 mm NaCl). Cell-free extracts prepared from strain DJ0035, DJ0077, or DJ0141 (Table 1) were applied to the appropriate affinity column and washed with 10 column volumes of anoxic buffer C, and samples were eluted using buffer C having 50 mm biotin. Excess bait protein and other proteins not specifically attached to MoFe protein subunits were removed by size-exclusion chromatography by loading 50 μl of sample onto a Superose 12^TM^ 10/300 GL column attached to an AKTA FPLC (GE Healthcare) equilibrated with buffer C and run at a flow rate of 0.5 ml/min.

### Alkylation of the MoFe protein α-Cys^275^ residue

Susceptibility of the MoFe protein α-Cys^275^ residue to alkylation by using I-AEDANS was performed as described by Christiansen *et al.* (18). Samples shown in Fig. 6*C* were treated with I-AEDANS for 15 s before quenching with 1 mm DTT.

### Spectroscopy

UV-visible spectra were collected on a Varian Cary 50 spectrophotometer. The samples were sealed inside a 1-cm path-length quartz cuvette under anoxic conditions. Continuous-wave X-band EPR spectra were recorded using a Bruker ESP-300 spectrometer with an EMX PremiumX microwave bridge and an EMX^PLUS^ standard resonator in perpendicular mode, equipped with an Oxford Instruments ESR900 continuous helium flow cryostat using VC40 flow controller for helium gas. Spectra were recorded at the following conditions: temperature, ∼12 K; microwave frequency, ∼9.38 GHz; microwave power, 20 milliwatts; modulation frequency, 100 kHz; modulation amplitude, 8.14 G; time constant, 20.48 ms. If not specified, each spectrum is the sum of five scans and is presented in this work after subtracting the cavity background signal recorded with an EPR tube with frozen 200 mm MOPS buffer. Final spectra were normalized to 43.5 μm MoFe protein based on absolute α- and β-subunit concentrations in the sample as described below.

### Acetylene and proton reduction assays

Ethylene and hydrogen gas formed were detected by flame ionization or thermal conductivity detectors coupled to a Shimadzu GC-2010-plus gas chromatograph equipped with a Carboxen® 1010 plot column, respectively. Specific activity is defined as nmol of product formed/min/mg of Strep-tagged MoFe protein. The assays were performed in 9-ml serum vials sealed with serum stoppers under the atmospheres indicated in Table 2. For assays involving Strep-tagged MoFe protein prepared from mutant strains, the reaction mixture included 100 μg of Strep-tagged MoFe protein and 1 mg of Fe protein prepared from DJ0033, whereas for assays involving Strep-tagged WT MoFe protein, 15 μg of MoFe protein and 165 μg of Fe protein were included. All assays included 5 mm Na_2_S_2_O_4_, 50 μg/ml creatine phosphokinase, 8 mm MgCl_2_, 1.35 mm ATP, and 18 mm phosphocreatine. Assay samples were incubated at 30 °C with agitation for 30 min or 1 h and were terminated by the injection of 100 μl of 4 n NaOH.

### Protein identification from SDS-PAGE bands via MALDI-TOF/TOF analysis

Gel bands were excised from SDS-polyacrylamide gels and destained using a 1:1 mixture of 50 mm ammonium bicarbonate (AmBic) plus LC/MS-grade acetonitrile. Destained gel pieces were dehydrated using LC/MS-grade acetonitrile and treated sequentially with 10 mm DTT in 50 mm AmBic for 1 h at 37 °C, 50 mm iodoacetamide in 50 mm AmBic for 30 min at room temperature in the dark, and 100 mm DTT in 50 mm AmBic to quench unreacted iodoacetamide. After washing the gel pieces once more with the destaining solution and dehydrating them using LC/MS-grade acetonitrile, just enough 10 ng/μl trypsin in 50 mm AmBic was added to cover the gel pieces, and the samples were incubated overnight at 37 °C.

The following day, 80:20 LC/MS-grade acetonitrile plus LC/MS-grade water supplemented with 0.1% (v/v) formic acid was added at the same volume as that of the trypsin solution the previous day. The samples were incubated in a sonicating water bath for 10 min, and then 1 μl of each sample was spotted onto a MALDI target plate. After the samples had dried, 1 μl of matrix solution (2 mg/ml α-cyano-4-hydroxycinnamic acid in 50:50 LC/MS-grade acetonitrile plus LC/MS-grade water supplemented with 0.1% (v/v) TFA) was added. Once the matrix dried, samples were analyzed using a 4800 MALDI-TOF/TOF (AB Sciex), first in positive ion reflector mode to obtain peptide masses and then in positive ion MSMS1kV mode to obtain tandem MS (MS2) spectra of the 12 most intense peaks observed in the MS1.

The resultant peak lists were searched against the combined Uniprot and NCBInr protein databases using the Mascot (Matrix Science) web server. Search parameters used were trypsin specificity with the possibility of one missed cleavage, a precursor tolerance of 250 ppm, a product tolerance of 0.25 Da, carbamidomethylation of cysteine residues as a fixed modification, and oxidation of methionine and pyroglutamate formation of glutamine residues found at the N terminus of a peptide as variable modifications. Proteins were confidently identified when at least three unique peptides were found or if at least one peptide with a Mascot score above 50 was identified and manually validated.

### Absolute MoFe protein quantification

Because MoFe prepared from different samples also included biotin-binding proteins, such as acetate carboxylase and pyruvate carboxylase, present in various concentrations depending on the sample (Fig. 6), as well as various assembly factors, absolute MoFe protein concentrations were estimated by MS (53). Briefly, six oligopeptides (NifD-LIDEVETLFPLNK, NifD-EEVESLIQEVLEVYPEK, NifD-GVSQSLGHHIANDAVR; and NifK-INIVPGFETYLGNFR, NifK-EEVESLIQEVLEVYPEK, NifK-AVDAILAASPYGK) were prepared as isotopically labeled synthetic peptides having a C-terminal Q-tag (JPT Peptide Technologies GmbH, Berlin, Germany). The peptides were solubilized in 0.1 m AmBic/acetonitrile (80:20, v/v) and combined. Samples for MoFe protein quantification were diluted to 1 mg/ml protein (50 mm Tris-HCl, pH 7.8) and combined with the standard mix at three different protein/standard ratios for analysis. Mixes were reduced, alkylated, treated with trypsin, and subsequently acidified and diluted 2-fold with 0.1% (v/v) formic acid in water.

Samples were analyzed using an Orbitrap Fusion Lumos mass spectrometer equipped with an Easy-nLC 1200 UPLC and an Easy Spray nanospray source (Thermo Scientific). The column utilized for peptide separation was a PepMap C18 (3 m, 100 Å, 75 μm × 15 cm; Thermo Scientific) in-line with an Acclaim PepMap 100 trapping column (100 μm × 2 cm; Thermo Scientific). Analyses utilized a 14-min gradient from 4 to 50% solvent B, where A was 0.1% (v/v) formic acid in water and B was 20:80 water/acetonitrile containing 0.1% (v/v) formic acid. The column temperature was maintained at 55 °C, and the ion transfer tube was maintained at 275 °C. Other mass spectrometer conditions were as follows: 2-μl injection, 400-nl/min flow rate, 2200-V ion spray voltage, MS scans utilizing the Orbitrap at 60,000 resolution for *m*/*z* 400–1200, an automatic gain control target of 2E5 with 100-ms maximum inject time. Profile data were collected in positive ion mode with the RF lens maintained at 30% of maximum.

Data were processed and analyzed using Xcalibur version 4.0 (Thermo Scientific), searching a 60-s window centered on the expected retention times and determining peak area for the expected *m*/*z* ± 0.04 using the genesis peak detection algorithm. Molar amounts of each peptide were determined by multiplying the ratio of light to heavy peptide with the amount of synthetic peptide injected and adjusting for the dilution of the original sample. Samples at each protein/standard ratio were run in triplicate. The values were averaged, and then the averages of each protein/standard ratio were averaged to yield the final result.

## Author contributions

E. J.-V. and D. R. D. conceptualization; E. J.-V. and D. R. D. formal analysis; E. J.-V., Z.-Y. Y., W. K. R., C. E.-E., and V. L. C. investigation; E. J.-V. methodology; E. J.-V. and D. R. D. writing-original draft; L. M. R. and L. C. S. writing-review and editing; D. R. D. funding acquisition; D. R. D. project administration.

## Footnotes

4 The abbreviations used are:

IMAC: immobilized metal-affinity chromatography
I-AEDANS: [2-(iodoacetamido)ethylamino]naphthalene-1-sulfonic acid
AmBic: ammonium bicarbonate.
